# Engineered delivery of Chinese herbal medicine-derived therapeutics for diabetic wound healing: a focus on NF-κB cascade modulation

**DOI:** 10.3389/fbioe.2026.1977317

**Published:** 2026-10-22

**Authors:** Boxun Chen, Qing Wang, Jialun Zhang, Xiaoshan Li, Ning Wang, Meiling Zou, Zhichao Huang, Jiayue Feng, Guangheng Zhang, Shang Ju

**Affiliations:** 1 Beijing University of Chinese Medicine, Beijing, China; 2 Dongzhimen Hospital, Beijing University of Chinese Medicine, Beijing, China; 3 Chengdu University of Traditional Chinese Medicine, Chengdu, China

**Keywords:** biomaterials, Chinese herbal medicine-derived therapeutics, diabetic wounds, engineered delivery, local exposure, NF-κB, tissue repair

## Abstract

Diabetic wounds are chronic, difficult-to-heal injuries of the skin and soft tissues in people with diabetes. Their impaired healing involves metabolic disturbances, oxidative stress, vascular and neural injury, immune dysregulation, and dysfunction of reparative cells. Nuclear factor kappa B (NF-κB) links metabolic stress, immune responses, and tissue repair. Its persistent pathological activation can maintain wound inflammation and impede the transition to the reparative phase, representing a key mechanism underlying impaired healing in diabetic wounds. Chinese herbal medicine-derived therapeutics can modulate upstream NF-κB stimuli, the core cascade, and downstream inflammatory output, but their local application is limited by poor dispersibility or chemical stability, washout by wound exudate, and tissue barriers. Engineered delivery can improve the dispersibility or formulation stability of these therapeutics and modulate their *in vitro* release. A small number of studies have further evaluated local delivery processes such as cellular uptake, but direct assessment of local exposure within the wound remains limited. This review examines how engineered delivery addresses delivery barriers and alters the local application of Chinese herbal medicine-derived therapeutics in diabetic wounds, while using the depth of NF-κB-related evidence as an important dimension for evaluating the reported biological effects. Existing studies show that the reparative effects of engineered formulations are often accompanied by attenuation of upstream NF-κB stimuli or reduced downstream inflammatory output, whereas only a few studies have further detected reduced phosphorylation of IκBα or p65. However, a complete causal evidence chain linking material properties, local exposure, the core IκB kinase (IKK)–IκBα–p65 cascade, and wound-healing outcomes has not yet been established. Future studies should align local delivery processes with the temporal and cellular distribution of aberrant NF-κB activation and strengthen the evaluation of therapeutic attribution, critical quality attributes, and biocompatibility. The goal of engineered delivery of Chinese herbal medicine-derived therapeutics is not to suppress NF-κB continuously, but to limit its persistent pathological activation and promote the transition of wounds from the inflammatory phase to the tissue repair phase.

## 1 Introduction

Diabetic wounds are injuries to the skin and soft tissues that occur in the setting of diabetes-related metabolic abnormalities and fail to heal normally because of associated vascular, neural, and immune pathological changes (Falanga, 2005; Baltzis et al., 2014). The global number of people with diabetes is approaching 600 million and continues to increase (Duncan et al., 2025). Diabetic foot ulcers are among the most serious clinical forms of diabetic wounds. The lifetime risk of developing a foot ulcer among people with diabetes is approximately 19%–34%, and the recurrence rate remains high after healing (Armstrong et al., 2023). When wounds are complicated by ischemia, infection, or tissue necrosis, patients may face risks of amputation and death. The 5-year mortality rate among patients with severe diabetic foot complications can be comparable to that of some malignancies (Armstrong et al., 2020).

Wound healing comprises hemostatic, inflammatory, proliferative, and remodeling phases that overlap and are dynamically coordinated. Nuclear factor kappa B (NF-κB) regulates the expression of cytokines, chemokines, and molecules involved in matrix remodeling and participates in early immune defense and the initiation of repair (Wilkinson and Hardman, 2020; Raziyeva et al., 2021). In diabetic wounds, persistent hyperglycemia, oxidative stress, necrotic tissue, and infection-related signals repeatedly relieve IκBα-mediated inhibition of NF-κB, preventing inflammatory transcription from subsiding promptly after the early defense response has been completed (Mirza et al., 2014; Theocharidis et al., 2020). Sustained NF-κB activation alters the local cytokine network within the wound, maintaining elevated levels of pro-inflammatory cytokines such as TNF-α, IL-1β, and IL-6 and thereby prolonging inflammation and aggravating tissue damage (Eming et al., 2017). This persistent NF-κB output in turn drives pro-inflammatory states in immune cells, inflammasome activation, damage to reparative cells, and extracellular matrix degradation, constituting an important mechanism underlying the impaired transition of diabetic wounds from the inflammatory phase to the proliferative and remodeling phases (Roy et al., 2022; Asghariazar et al., 2025).

Current standard treatments can address some local factors that impede wound healing, yet some diabetic wounds remain unhealed for prolonged periods (Sidhu and Harbuzova, 2024). Persistent metabolic stress and dysregulated inflammatory responses can sustain immune imbalance and impair the function of keratinocytes, fibroblasts, and endothelial cells, further hindering tissue repair (Wang et al., 2025). Therefore, in addition to addressing adverse local factors, modulating the inflammatory microenvironment is an important approach to promoting diabetic wound healing. Chinese herbal medicine-derived therapeutics have antioxidant, inflammation-modulating, and tissue-repair-promoting activities, providing a potential approach to improving the diabetic wound microenvironment (Zhou et al., 2022). However, poor dispersibility or stability of some therapeutics, together with wound exudate and tissue barriers, can limit their local effects. Engineered delivery can use material architecture and formulation processes to improve the dispersibility and stability of these therapeutics, modulate their release, and reduce diffusive loss or washout by wound exudate, thereby improving formulation performance and the local delivery process.

In recent years, reviews have summarized advances in this field from perspectives including the modulation of NF-κB by plant-derived bioactive compounds and the use of Chinese medicine in combination with nanotechnology and biomaterials to promote wound repair (Yadav et al., 2024; Priyadharshini and Sabarees, 2026). However, existing studies have largely focused on either the wound-healing mechanisms of bioactive therapeutics or engineered materials themselves. Less attention has been paid to how engineered delivery affects the local delivery of Chinese herbal medicine-derived therapeutics and how these delivery-related changes relate to NF-κB cascade modulation. Accordingly, this review examines how engineered delivery alters the local application of Chinese herbal medicine-derived therapeutics in diabetic wounds, using persistent pathological NF-κB activation as a mechanistic framework for evaluating the associated inflammatory and reparative effects. It compares the delivery functions, advantages, and limitations of different strategies and evaluates how these changes relate to local exposure and wound repair. NF-κB-related evidence is considered according to whether it reflects upstream stimuli, the core IκB kinase (IKK)–IκBα–p65 cascade, or downstream inflammatory outputs. By identifying where the links among material performance, local exposure, NF-κB modulation, and wound repair remain incomplete, this review aims to support the rational design and translational evaluation of these delivery systems.

## 2 Literature search and review scope

PubMed and Web of Science were searched for relevant English-language literature published or formally accepted for publication up to 30 July 2026. Topic-based searches, reference tracing, and targeted additional searches were used to identify additional relevant articles. Search terms covered diabetic wounds, Chinese herbal medicine-derived therapeutics, engineered delivery, and the NF-κB cascade, including “diabetic wound,” “diabetic foot ulcer,” “traditional Chinese medicine,” “Chinese herbal medicine,” “phytochemical,” “polysaccharide,” “herbal extract,” “plant-derived extracellular vesicle,” “biomaterial,” “drug delivery,” “hydrogel,” “nanoparticle,” “nanofiber,” “microneedle,” “NF-kappaB,” “IKK,” “IκBα,” and “RelA/p65.” Search terms were combined across topics, with additional keywords added according to specific therapeutics, material types, and signaling molecules. Only peer-reviewed journal articles were considered; unpublished data, preprints, submitted manuscripts, conference abstracts, and personal communications were excluded.

This review primarily included studies of engineered delivery of Chinese herbal medicine-derived therapeutics for diabetic wounds and examined how material design affected formulation performance, local delivery, and NF-κB-related effects. Studies of free therapeutics were used mainly to characterize the pharmacological basis for their modulation of NF-κB. Conventional topical formulations were included only when they showed local effects in diabetic wounds and provided NF-κB-related evidence. For composite systems containing additional active components, when component-specific controls were lacking, the observed effects were attributed to the final formulation rather than to any individual component. Studies in normal wounds were used mainly to provide mechanistic context for NF-κB, whereas non-engineered studies were not considered direct evidence of delivery-related benefit. Study-level formulation design, delivery evidence, experimental context, NF-κB-related readouts and evidence levels, and wound-repair outcomes are summarized in Table 1.

**TABLE 1:** Engineered delivery of Chinese herbal medicine-derived therapeutics in diabetic wound models: formulation and delivery evidence, depth of NF-κB-related evidence, and repair outcomes.

Therapeutic/study	Platform/role	Delivery evidence	Model/comparator(s)	NF-κB-related readout	Evidence level	Repair outcome
Defined small-molecule therapeutics						
Berberine (Panda et al., 2021)	Lecithin-chitosan NPs (cargo)	168.4 nm; EE 82.3% Sustained release *in vitro*	Diabetic rat wounds Free berberine; blank NPs	Inflammatory-cell infiltration ↓	Indirect	Closure accelerated Angiogenesis/mature collagen ↑
Berberine (Zhang et al., 2020)	Berberine nanocolloids in Ca^2+^-PVA/alginate hydrogel (cargo)	Slower release *in vitro*	Diabetic rat wounds Hydrogel controls	Total NF-κB ↓ TNF-α/IL-6 ↓	Expression/pathway	Closure accelerated Angiogenesis/remodeling ↑
Berberine (Wang Q. et al., 2024)	Radially aligned PHBV fibers (cargo)	Gradual release for ∼1 month *in vitro* Directional fibroblast migration	Diabetic wounds Fiber controls	Macrophage IL-6/TNF-α ↓	Indirect	Closure accelerated Tissue repair ↑
Berberine-ZnO system (Yin et al., 2022)	ZnO nanocolloids in injectable hydrogel (multicomponent)	Stable incorporation in gel network	Diabetic wounds Hydrogel controls	Nrf2/HO-1/NQO1 ↑ Pro-inflammatory mediators ↓	Indirect	Closure accelerated Tissue repair ↑
Curcumin (Li et al., 2019)	Chitosan NPs (cargo)	Slower release *in vitro* Macrophage uptake ↑	Diabetic rat wounds + macrophages Free curcumin; blank NPs	Wound inflammation ↓	Indirect	Closure accelerated Angiogenesis ↑
Curcumin (Liao et al., 2021)	Heparin-grafted aligned PLGA fibers (cargo)	Sustained release *in vitro* Directional migration; growth-factor retention	Diabetic wounds Fiber controls	NF-κB mRNA ↓ Glutathione peroxidase ↑	Expression/pathway	Re-epithelialization ↑ Angiogenesis/collagen ↑
Quercetin (Liu et al., 2026)	Chitosan NPs in GelMA (cargo)	222.0 ± 7.57 nm; EE 98.55% ± 0.56% Release: 8 → ∼48 h *in vitro*	Diabetic mouse wounds + high-glucose cells Free quercetin/GelMA; blank hydrogel	Total NF-κB/RAGE ↓ ROS/inflammatory mediators ↓	Expression/pathway	Closure accelerated Angiogenesis/collagen ↑
Taxifolin (Wang Y. et al., 2023)	Alginate/PVA electrospun fibers (cargo)	Continuous fibers Release: 64.6% ± 3.7%/24 h *in vitro*	Diabetic wounds Nanofiber controls	TLR4/NF-κB/NLRP3-associated proteins ↓	Expression/pathway	Closure accelerated Angiogenesis/repair ↑
Glycyrrhizic acid (Qian et al., 2022)	GA-Zn^2+^/silk fibroin hydrogel (therapeutic + structural)	Injectable; wound-conforming Structural stability ↑	Diabetic wounds Hydrogel controls	Macrophage-state remodeling Inflammatory → reparative transition	Indirect	Closure accelerated Tissue regeneration ↑
Glycyrrhizic acid (Li J. et al., 2025)	GA-Zn^2+^/methacrylated konjac glucomannan (therapeutic + structural)	Hydrogel stability ↑	Diabetic mouse wounds Hydrogel controls	ROS/TNF-α ↓ Reparative macrophage phenotype ↑	Indirect	Re-epithelialization ↑ Angiogenesis/collagen ↑
Ginsenoside Rg5 (Shen et al., 2023)	F127-PEI NPs in polysaccharide-collagen hydrogel (cargo)	Dispersion ↑; prolonged release *in vitro* Shape adaptation to tunneling wounds	Tunneling diabetic wounds Unloaded-hydrogel controls	Not assessed	None	Healing time ↓ Tissue repair ↑
Paeoniflorin-ZnO system (Guo et al., 2022)	ROS-responsive micelles + ZnO in Schiff-base hydrogel (multicomponent)	Acid/ROS-responsive release *in vitro*	Infected diabetic wounds Component-control hydrogels	Not assessed	None	Infection controlled Angiogenesis/repair ↑
Rhein self-assemblies (Zhao et al., 2020)	Rhein fibers in β-CD/ferrocene hydrogel (therapeutic + self-assembling)	ROS-triggered release Assembly integrity preserved	Chronic diabetic wounds Free rhein; hydrogel controls	ROS ↓ Excessive inflammation ↓	Indirect	Closure accelerated Tissue repair ↑
Rhein-CGRP system (Zhou et al., 2026)	Rhein/GelMA + CGRP (multicomponent)	Rhein/CGRP release ∼10 days *in vitro* Adhesion/mechanical stability ↑	Diabetic wounds GelMA; rhein-GelMA; CGRP-containing groups	NF-κB/NET pathway enrichment ↓ROS/MPO/CitH3 ↓	Expression/pathway	Closure accelerated Angiogenesis/reconstruction ↑
Cinnamaldehyde (Lin et al., 2025)	MMP-9-responsive injectable hydrogel (cargo)	Progressive release under elevated MMP-9	Diabetic wounds + high-glucose endothelial cells Hydrogel controls	Not assessed	None	Endothelial migration/angiogenesis ↑ Closure accelerated
Herbal polysaccharide-based therapeutics						
Oxidized *Ganoderma lucidum* polysaccharide (Li F. et al., 2024)	Double-network hydrogel (therapeutic + crosslinking)	Aldehyde 1.33 ± 0.05 mmol/g Oxidation 32.28% ± 2.55%; stability/adhesion ↑	Diabetic wounds + high-glucose cells Hydrogel controls	ROS ↓ Reparative macrophage phenotype ↑	Indirect	Epidermal/collagen architecture restored Repair accelerated
*Astragalus membranaceus* polysaccharide-baicalein-berberine system (Liu et al., 2024)	ROS-responsive NPs in dissolving microneedles (responsive carrier + cargo)	Wound penetration/dissolution ROS-triggered NP disassembly	Biofilm-covered diabetic wounds Component-control microneedles	p65 phosphorylation ↓ TNF-α/IL-6/ROS ↓	Direct	Closure accelerated Angiogenesis/collagen ↑
Single-herb extracts and fractions						
*Poria cocos* triterpene fraction (Ding et al., 2023)	Nanoemulsion in hyaluronic acid hydrogel (fraction as cargo)	Dispersion stability ↑ Uniform porous/elastic matrix	Diabetic rat wounds Formulation controls	Wound inflammation ↓ Reparative macrophage phenotype ↑	Indirect	Re-epithelialization ↑ Angiogenesis/collagen ↑
*Scutellaria baicalensis* extract (Zhu et al., 2024)	Extract-loaded self-healing hydrogel (extract as cargo)	Release: 68.8% ± 2.1%/12 h in PBS ROS-scavenging activity retained	*S. aureus*-infected diabetic wounds Free extract; blank hydrogel	Oxidative stress ↓ Wound inflammation ↓	Indirect	Closure accelerated Epidermal regeneration/collagen ↑
*Centella asiatica* extract (Wang X. et al., 2024)	3D-printed gelatin/alginate scaffold (extract as cargo)	Defined geometry Adaptable to wound shape	Chronic diabetic wounds Scaffold controls	Macrophage/local immune responses modulated	Indirect	Closure accelerated Tissue reconstruction ↑
Herbal formulas and extract combinations						
Huoxue Tongluo Formula extract (Chen et al., 2025)	Photocrosslinked hydrogel (multicomponent extract)	Interpenetrating network Mechanical properties/dispersion ↑	Infected diabetic wounds Hydrogel controls	Bacterial burden/ROS ↓ Inflammatory-cell infiltration ↓	Indirect	Epidermal regeneration ↑ Angiogenesis/collagen ↑
*Salvia miltiorrhiza*-*Pueraria lobata* extracts (Li Y. et al., 2024)	Gelatin/PLA nanoyarn dressing (multicomponent extracts)	Mechanical properties/wettability characterized Cell-supportive fibrous interface	Diabetic wounds Blank-dressing controls	Pro-inflammatory mediator release ↓	Indirect	Re-epithelialization ↑ Angiogenesis/hair follicles ↑
Dragon’s blood-*Salvia miltiorrhiza* extracts (Tang et al., 2024)	Dissolving PVP microneedles (dual-extract cargo)	Tissue penetration/dissolution Representative release quantified over 6 h	Diabetic wounds Single- vs. dual-extract microneedles	Not assessed	None	Cell migration/microcirculation ↑ Healing accelerated
Herbal-derived nanovesicles and related biological nanoparticles						
Ginseng root-derived exosome-like nanovesicles (Tan et al., 2024)	Natural nanovesicles (intact bioactive unit)	Particle characterization Endothelial-cell uptake	Diabetic ulcers + high-glucose endothelial cells Vehicle controls	Intracellular ROS ↓ Metabolism shifted toward glycolysis	Indirect	Angiogenesis ↑ Ulcer repair ↑
*Salvia miltiorrhiza*-derived exosome-like nanoparticles (Bai et al., 2026)	Natural nanoparticles (intact bioactive unit)	Particle-level characterization	Diabetic wounds + reparative-cell models Vehicle controls	ROS ↓ IL-1β/IL-6/TNF-α ↓	Indirect	Closure accelerated Tissue repair ↑
Turmeric-derived nanoparticles (Wu et al., 2024)	Cellulose nanofiber/alginate aerogel (particle cargo)	Storage stability ↑ Fluid absorption/conformity/release	Diabetic wounds + fibroblasts/macrophages Blank aerogel; particle controls	Nrf2/HO-1/NQO1 ↑ TLR4/MyD88/inflammatory mediators ↓	Indirect	Wound inflammation ↓ Tissue reconstruction ↑
*Aloe vera* peel-derived vesicle mimetics (Wang J. et al., 2026)	Modified HPMC hydrogel (vesicle-mimetic cargo)	Particle preservation > Carbomer gel Sustained release 72 h *in vitro*	Diabetic mouse wounds + LPS macrophages Free mimetics; blank hydrogel	Total NF-κB ↓ COX-2/iNOS ↓	Expression/pathway	Closure accelerated Re-epithelialization/collagen remodeling ↑
*Houttuynia cordata*-derived EVs (Yang et al., 2025)	Responsive collagen hydrogel (EV cargo)	Adhesive/self-healing High-glucose/ROS-responsive EV release	Diabetic wounds Blank hydrogel; free EVs	p-IκBα ↓; p-p65 ↓ IκBα restored; inflammatory mediators ↓	Direct	Closure accelerated Angiogenesis/collagen ↑
*Ginkgo biloba*-derived exosome-like nanovesicles (Wu et al., 2026)	ROS-responsive bilayer hydrogel (multicomponent)	Early antibacterial outer layer ROS-triggered inner-layer EV release	Infected diabetic wounds Component-control bilayer hydrogels	Infection-associated injury ↓ Inflammatory burden ↓	Indirect	Cell proliferation/angiogenesis ↑ Closure ↑
*Houttuynia cordata* carbon dots + EVs (Zhang et al., 2026)	Co-loaded GelMA (dual bioactive units)	Stable co-loading Single-component comparison	Infected diabetic wounds Single- vs. co-loaded hydrogels	ROS ↓ Reparative macrophage phenotype ↑	Indirect	Closure accelerated Tissue repair ↑
*Momordica charantia* nanovesicles + MSC exosomes (Weng et al., 2025)	GelMA/dopamine hydrogel (multivesicle system)	Sustained release of both vesicle populations	Diabetic mouse wounds Blank; single-exosome hydrogels	ROS ↓ Reparative macrophage phenotype ↑	Indirect	Closure accelerated Angiogenesis/collagen ↑

NF-κB, evidence level reflects the directness of NF-κB-related evidence in the corresponding study, not overall study quality or causal mediation of wound repair. Direct indicates core activation readouts, including IκBα phosphorylation/degradation, p65 phosphorylation or nuclear translocation, DNA, binding, or transcriptional activity. Expression/pathway indicates NF-κB mRNA, total NF-κB/p65 protein, or NF-κB-related protein-level or omics-based pathway changes without direct assessment of activation. Indirect indicates only upstream or downstream inflammatory, oxidative, or immune-related readouts. None indicates that NF-κB-related endpoints were not assessed. Evidence from separate non-engineered studies was not used to upgrade the evidence level. For multicomponent formulations, effects are attributed to the complete formulation unless component-specific controls support attribution. *In vitro* release or material-performance data were not considered direct evidence of *in vivo* wound exposure. ↑, increased; ↓, decreased.

β-CD, β-cyclodextrin; CGRP, calcitonin gene-related peptide; CitH3, citrullinated histone H3; EE, encapsulation efficiency; EVs, extracellular vesicles; GA, glycyrrhizic acid; GelMA, gelatin methacryloyl; HPMC, hydroxypropyl methylcellulose; MPO, myeloperoxidase; MSC, mesenchymal stem cell; NETs, neutrophil extracellular traps; NF-κB, nuclear factor kappa B; NPs, nanoparticles; PBS, phosphate-buffered saline; PEI, polyethyleneimine; PHBV, poly (3-hydroxybutyrate-co-3-hydroxyvalerate); PLA, polylactic acid; PLGA, poly (lactic-co-glycolic acid); PVA, poly (vinyl alcohol); PVP, polyvinylpyrrolidone; ROS, reactive oxygen species.

## 3 Overview of NF-κB signaling

### 3.1 NF-κB family members and structural features

NF-κB is a family of transcription factors widely involved in inflammation, immune regulation, cell proliferation, apoptosis, and tissue repair (Liu et al., 2017). The mammalian NF-κB family comprises five major members: RelA/p65, RelB, c-Rel, NF-κB1 p105/p50, and NF-κB2 p100/p52, all of which share a highly conserved N-terminal Rel homology domain that mediates DNA binding, dimerization, nuclear localization, and interactions with IκB proteins. RelA/p65, RelB, and c-Rel contain transactivation domains, whereas p50 and p52 lack typical transactivation domains and depend on dimer composition and co-regulators for transcriptional activity (Guo et al., 2024). NF-κB1 and NF-κB2 are synthesized as the precursors p105 and p100 and are processed to p50 and p52, respectively (Oeckinghaus and Ghosh, 2009). Among these, the RelA/p65–p50 heterodimer is the predominant active form of the canonical NF-κB pathway and mediates much of its inflammation-related transcriptional activity (Hayden and Ghosh, 2012).

### 3.2 Canonical and non-canonical activation of NF-κB signaling

The canonical NF-κB pathway is typically activated by TNF-α, IL-1β, lipopolysaccharide (LPS), and damage-associated signals. At rest, the RelA/p65–p50 heterodimer is retained in the cytoplasm by IκBα. Receptor signaling activates the IκB kinase (IKK) complex, which contains IKKα, IKKβ, and NEMO/IKKγ. IKKβ phosphorylates IκBα, promoting its ubiquitination and proteasomal degradation (Mercurio et al., 1997). RelA/p65–p50 then translocates to the nucleus, binds κB DNA response elements, and induces genes involved in inflammation, immune responses, and cell survival (Mao et al., 2025).

The non-canonical pathway is activated by a more restricted set of receptors, including BAFFR, CD40, RANK, and LTβR, and proceeds through NF-κB-inducing kinase (NIK), IKKα-dependent processing of p100 to p52, and nuclear translocation of RelB–p52 (Coope et al., 2002). It has slower activation kinetics and is more closely associated with lymphoid development and adaptive immune regulation (Senftleben et al., 2001). The non-canonical pathway remains relatively understudied in diabetic wounds, while available evidence involving Chinese herbal medicine-derived therapeutics mainly concerns canonical NF-κB signaling.

### 3.3 Transcriptional output and network regulation of the NF-κB cascade

The biological effects of NF-κB depend on Rel dimer composition, κB DNA response-element sequences, and cellular context. NF-κB activation also induces negative-feedback regulators, including IκBα and A20. Newly synthesized IκBα limits nuclear RelA/p65 activity, whereas A20 attenuates receptor-proximal signaling. Together, these feedback mechanisms shape the magnitude and duration of NF-κB activation (Werner et al., 2008). NF-κB function therefore depends not only on the extent of activation but also on its temporal pattern and cellular context.

NF-κB also interacts with signaling pathways involved in oxidative stress, inflammasome regulation, and tissue repair. Signaling pathways such as phosphoinositide 3-kinase (PI3K) can influence IKK–NF-κB activity, whereas nuclear factor erythroid 2-related factor 2 (Nrf2), in addition to mediating antioxidant responses, can limit the transcription of pro-inflammatory genes such as IL-6 and IL-1β (Kobayashi et al., 2016; Zhang et al., 2024). NF-κB also participates in the priming of the NLRP3 inflammasome by inducing NLRP3 and pro-IL-1β expression, thereby enabling cells to respond to subsequent activation signals. In some contexts, NF-κB can also limit excessive inflammasome activation by promoting the clearance of damaged mitochondria (Zhong et al., 2016). Crosstalk between NF-κB and repair-related pathways such as HIF-1α/VEGF and TGF-β/Smad also affects angiogenesis, extracellular matrix deposition, and tissue remodeling (Kamal et al., 2024). Changes in inflammatory mediators, NF-κB mRNA, or total NF-κB protein alone cannot establish pathway activation or inhibition. More direct evidence can be obtained by assessing IκBα degradation, p65 phosphorylation and nuclear translocation, DNA binding, or NF-κB transcriptional activity.

## 4 NF-κB cascade regulation in impaired diabetic wound healing

### 4.1 Dynamic regulation of NF-κB during normal wound healing

During normal wound healing, NF-κB activation shows a distinct temporal pattern, rising rapidly during the early phase of injury and gradually declining as inflammation resolves (Samulevich et al., 2024). Early after tissue injury, damage-associated molecular patterns and locally produced inflammatory mediators such as TNF-α and IL-1β can activate NF-κB signaling in cells surrounding the wound and in infiltrating immune cells. Activated NF-κB in turn induces the expression of cytokines, chemokines, and adhesion molecules, promotes inflammatory cell recruitment, and contributes to the clearance of necrotic tissue and pathogens (Hong et al., 2023). Chen et al. found that Prx1-lineage fibroblasts exhibited higher NF-κB activity during the early phase of injury. Specific deletion of *Ikbkb* in this cell population reduced macrophage recruitment and bacterial clearance within wounds and markedly delayed the healing of both oral and skin wounds (Chen et al., 2024). These findings suggest that moderate NF-κB activation early after injury does not merely promote inflammation, but also contributes to immune defense and the initiation of repair.

As pathogens and necrotic tissue are progressively cleared, the focus of wound healing shifts from inflammatory defense to cell proliferation and tissue reconstruction, and NF-κB transcriptional output must decline accordingly. Shen et al. found that injury induced phosphorylation of α-catenin at Ser641 in fibroblasts. This modification limited NF-κB activation by stabilizing IκBα and promoted fibroblast proliferation, migration, and skin wound healing (Shen et al., 2022). Normal wound healing therefore depends on dynamic, phase-specific regulation of NF-κB rather than sustained enhancement or unidirectional suppression of the pathway.

### 4.2 Persistent NF-κB activation driven by the diabetic wound microenvironment

Unlike the transient and controlled NF-κB response in normal wounds, NF-κB transcriptional activity in diabetic wounds fails to subside promptly after the onset of inflammation. Persistent hyperglycemia, oxidative stress, and tissue injury signals repeatedly activate IKK, promoting IκBα phosphorylation, ubiquitination, and proteasomal degradation and thereby sustaining nuclear translocation of RelA/p65–p50 (Qin and Deng, 2025). Compared with nondiabetic wounds, human diabetic wounds show increased expression of TLR2/4, MyD88, and IRAK1, accompanied by enhanced p65 DNA-binding activity (Dasu and Martin, 2014). Thus, aberrant signaling in diabetic wounds is not confined to upstream receptors and adaptor proteins but extends to NF-κB activity at the nuclear transcriptional level.

Persistent hyperglycemia is an important metabolic driver of this process. Hyperglycemia promotes reactive oxygen species (ROS) generation by activating protein kinase C (PKC) α/δ and NADPH oxidase, while also upregulating TLR2/4 expression. Enhanced TLR2/4–MyD88–IRAK1 signaling further promotes IKK activation and RelA/p65 nuclear translocation (Portou et al., 2020). Dasu et al. found that inhibition of PKC or NADPH oxidase attenuated hyperglycemia-induced TLR2/4 expression and NF-κB activation (Dasu et al., 2008). TLR2 deficiency reduced MyD88 and IRAK1 phosphorylation and NF-κB activity, while accelerating wound closure in diabetic mice (Dasu et al., 2010).

Hyperglycemia also accelerates the accumulation of advanced glycation end products (AGEs). AGEs continue to accumulate in the slowly renewing extracellular matrix and activate the receptor for advanced glycation end products (RAGE). RAGE can transmit signals through TLR2/4-associated adaptor proteins and promote ROS generation via NADPH oxidase, thereby accelerating IκBα degradation and RelA/p65 nuclear translocation (Sakaguchi et al., 2011). Once in the nucleus, NF-κB can also bind the *AGER* promoter and increase RAGE expression, sustaining cellular responsiveness to AGEs and establishing an AGEs–RAGE–NF-κB positive feedback loop (Tanaka et al., 2000). Excessive ROS can also cause mitochondrial damage and the release of mitochondrial DNA (mtDNA), which in turn activates cyclic GMP-AMP synthase–stimulator of interferon genes (cGAS–STING) signaling. Geng et al. found markedly increased levels of cGAS, STING, and p-p65 in human diabetic wounds, while cGAS–STING signaling in diabetic mouse wounds also failed to decline as healing progressed (Geng et al., 2023). cGAS–STING may therefore provide another upstream input linking mitochondrial damage to sustained NF-κB activation. Together, hyperglycemia, AGE accumulation, and mitochondrial injury generate mutually reinforcing inflammatory signals that prevent NF-κB activity from declining promptly after the early defense response.

### 4.3 NF-κB-mediated persistent inflammation and immune dysregulation

Sustained NF-κB activation prevents the inflammatory response in diabetic wounds from resolving promptly and alters the functional states of macrophages and neutrophils, thereby perpetuating local immune dysregulation. Macrophages are key effector cells in this process. Although wound macrophages occupy a complex continuum of functional states, pro-inflammatory programs can remain sustained in the diabetic environment, accompanied by persistent expression of pro-inflammatory markers such as IL-1β, TNF-α, and iNOS (Mirza et al., 2013). Sustained NF-κB activation may contribute to this persistence. Continued TNF-α production by macrophages can further sustain NF-κB-related inflammatory transcription, making local inflammation more difficult to resolve (denDekker et al., 2020). Meanwhile, persistent NF-κB activity induces continued expression of chemokines such as CXCL1, CXCL2, and CXCL8, promoting ongoing neutrophil recruitment to the wound. The diabetic environment also increases the propensity for neutrophil extracellular trap (NET) formation, leading to excessive NET accumulation within the wound (Wong et al., 2015).

NETs not only reflect excessive neutrophil activation but can also further amplify macrophage inflammation. Components of NETs, including DNA and histones, can themselves act as strong inflammatory stimuli (Tsourouktsoglou et al., 2020). In diabetic wounds, Liu et al. further found that excessive NETs upregulated NLRP3 and pro-IL-1β through TLR4/9–NF-κB signaling and promoted NLRP3 inflammasome activation in macrophages. Degradation of NETs by DNase I reduced inflammasome activation and inflammatory cell infiltration and accelerated wound healing (Liu et al., 2019). In a diabetic mouse wound model, Li et al. found that treatment with the specific NF-κB inhibitor Ro 106–9,920 reduced the expression of the NET markers CitH3 and PAD4, decreased levels of inflammasome-related proteins including NLRP3, ASC, and caspase-1, and markedly accelerated wound closure (Li H. et al., 2025). Taken together, these studies support a role for NF-κB in linking neutrophil NET formation with macrophage NLRP3 inflammasome activation, thereby aggravating immune dysregulation and delaying repair in diabetic wounds.

### 4.4 NF-κB-mediated dysfunction of reparative cells and aberrant tissue remodeling

Sustained NF-κB activation not only prolongs wound inflammation but also compromises the reparative functions of keratinocytes, fibroblasts, and vascular endothelial cells, thereby impairing re-epithelialization, granulation tissue formation, and angiogenesis. Hyperglycemia can further increase the loss of reparative cells by activating multiple regulated cell-death pathways. PANoptosis integrates mechanisms of pyroptosis, apoptosis, and necroptosis, thereby increasing both cell death and the release of inflammatory mediators (Sun et al., 2024). Wang et al. found that high glucose enhanced the interaction between TAK1 and p65, increased IκBα and p65 phosphorylation, and elevated levels of PANoptosis-related proteins, including p-RIPK1, cleaved caspase-1, and cleaved caspase-3. TAK1 silencing improved the proliferation and migration of keratinocytes and fibroblasts and enhanced the tube-forming capacity of endothelial cells. In diabetic mouse wounds, TAK1 silencing reduced PANoptosis-related changes, increased collagen deposition and microvessel formation, and accelerated wound closure (Wang X. et al., 2026). The findings implicate TAK1/NF-κB signaling in PANoptosis and suggest that increased death and dysfunction of reparative cells may contribute to impaired regeneration in diabetic wounds.

Aberrant tissue remodeling is also characterized by insufficient extracellular matrix synthesis and excessive matrix degradation. TGF-β1/Smad signaling promotes fibroblast activation and collagen synthesis, whereas sustained NF-κB activation can interfere with this reparative process. In a diabetic mouse wound model, Gong et al. found that recombinant TGF-β1 increased Smad2/3 phosphorylation and Smad4 expression and promoted wound closure. When NF-κB was activated, these responses were attenuated, Smad7 expression increased, and the wound-healing effect of TGF-β1 was reduced (Gong et al., 2022). NF-κB may also promote matrix degradation by increasing MMP-9 expression. Zhu et al. found that AGE-BSA enhanced NF-κB nuclear translocation in primary keratinocytes and increased both MMP-9 expression and enzymatic activity. Blocking upstream Notch1 signaling reversed these changes and, in diabetic rat wounds, reduced MMP-9 expression, increased collagen deposition, and accelerated wound closure (Zhu et al., 2022). These findings support the involvement of the Notch1/NF-κB/MMP-9 axis in matrix degradation, but direct transcriptional regulation has not yet been confirmed through p65-specific intervention. Sustained NF-κB activation can therefore aggravate both reparative cell dysfunction and matrix remodeling imbalance, ultimately impairing tissue reconstruction within the wound.

### 4.5 Post-transcriptional and epigenetic maintenance of aberrant NF-κB activation

In addition to repeated upstream stimulation, weakened negative-feedback regulation can also prevent NF-κB activity from declining promptly. MiR-146a limits signal transmission from TLRs and IL-1R to NF-κB by targeting IRAK1 and TRAF6 mRNAs. Xu et al. found reduced miR-146a expression in diabetic wounds, accompanied by increased expression of IRAK1, TRAF6, and related pro-inflammatory genes (Xu et al., 2012). Bi et al. further showed that miR-146a deficiency enhanced IRAK1-, TRAF6-, and NF-κB-related inflammatory responses, increased IL-1β and TNF-α expression and neutrophil infiltration, and delayed skin wound healing in both normal and diabetic mice (Bi et al., 2022). Importantly, this study primarily assessed NF-κB expression without evaluating p65 phosphorylation or nuclear translocation. These findings provide stronger evidence that loss of miR-146a-mediated negative feedback aggravates inflammation than direct evidence of sustained activation of the core NF-κB cascade. At the nuclear level, the long non-coding RNA Lethe can restrict RelA/p65 activity. Zgheib et al. found that high-glucose exposure reduced Lethe expression in macrophages while increasing nuclear p65 levels, NOX2 expression, and ROS production. Lethe overexpression reduced nuclear p65 without altering total p65 protein and also lowered NOX2 expression and ROS levels (Zgheib et al., 2017). Reduced Lethe expression and increased NOX2 were also observed in diabetic mouse wounds, although the effect of Lethe on healing outcomes was not tested through direct intervention within the wound. Together, reduced miR-146a and Lethe weaken restraint on receptor-proximal signaling and nuclear p65 activity, respectively, making NF-κB-driven inflammatory output more difficult to terminate.

Chromatin state further determines whether NF-κB target genes can transition from active transcription to repression. The histone methyltransferase SETDB2 increases the repressive histone mark H3 lysine 9 trimethylation (H3K9me3) at NF-κB-binding sites, thereby reducing chromatin accessibility at inflammatory genes (Schliehe et al., 2015). Kimball et al. found reduced SETDB2 expression and lower H3K9me3 levels at relevant promoter regions in macrophages from diabetic wounds. Myeloid-specific deletion of *Setdb2* further increased inflammatory gene expression, impaired the transition of macrophages toward a reparative state, and delayed wound healing (Kimball et al., 2019). In contrast to SETDB2, the histone demethylase JMJD3 removes the repressive histone mark H3 lysine 27 trimethylation (H3K27me3). Single-cell sequencing by Audu et al. revealed increased JMJD3 expression in macrophages from human diabetic wounds. During the later phase of wound healing in diabetic mice, persistently elevated IL-6 promoted JMJD3 expression through JAK1/3–STAT3 signaling and reduced H3K27me3 at the promoters of NF-κB-related inflammatory genes. Targeted inhibition of JMJD3 in macrophages reduced inflammatory mediators and STING expression and improved wound repair (Audu et al., 2022). Reduced SETDB2 limits the deposition of repressive marks, whereas elevated JMJD3 accelerates their removal, leaving NF-κB-related inflammatory genes in a more transcriptionally permissive chromatin state. Loss of non-coding RNA-mediated negative feedback and dysregulated chromatin repression do more than increase individual inflammatory mediators; they weaken the mechanisms that terminate NF-κB-driven inflammatory transcription. Interventions targeting these reversible regulatory mechanisms may help restore inflammatory restraint during the later reparative phase while preserving cell specificity and temporal control, thereby avoiding interference with the early defensive functions of NF-κB after injury. These temporal and cellular differences also provide a rationale for considering not only what is delivered to the wound, but where and for how long the therapeutic is locally available. The mechanisms underlying persistent pathological NF-κB activation in diabetic wounds are summarized in Figure 1.

**FIGURE 1:**
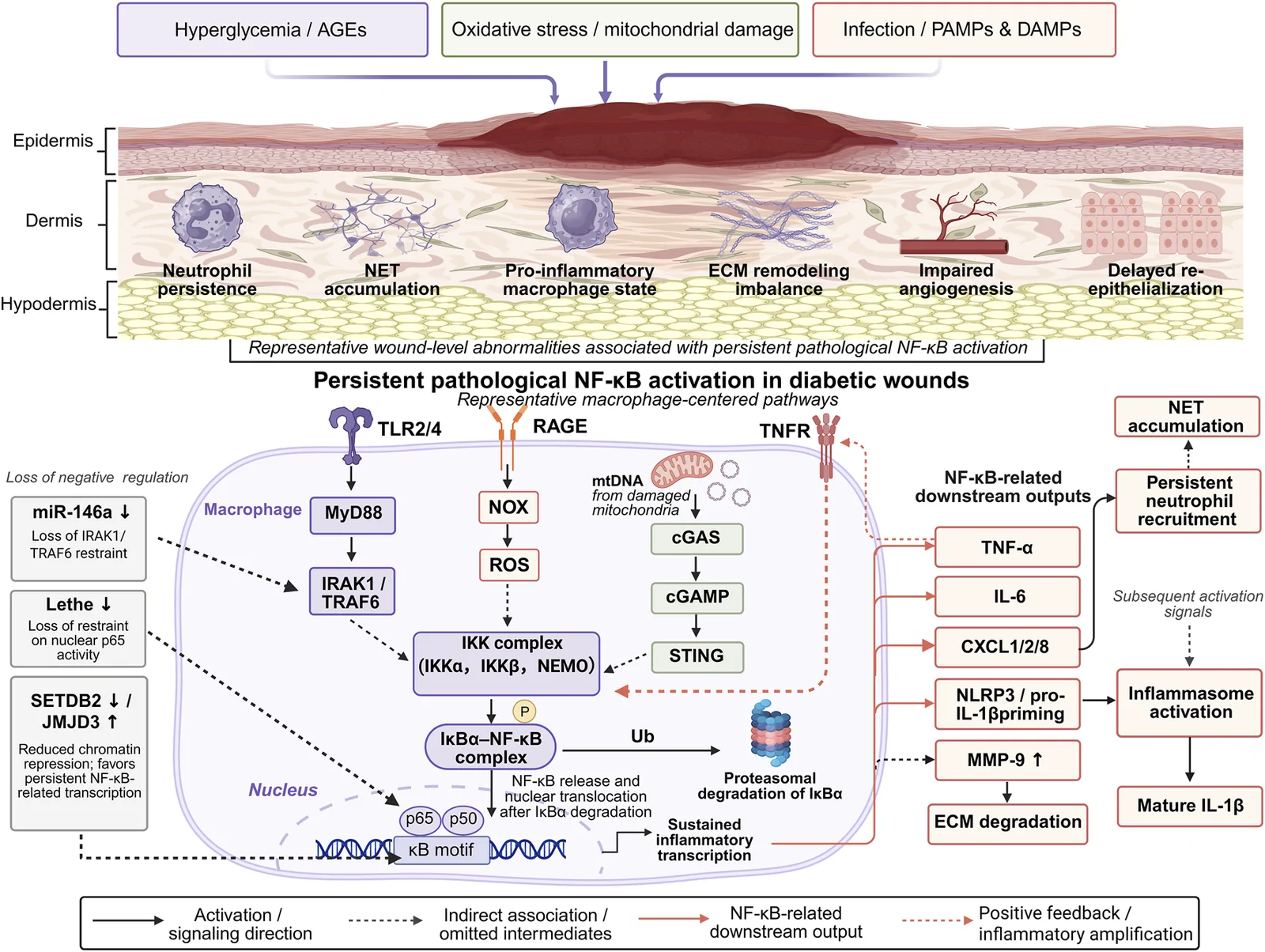
Persistent pathological NF-κB activation in diabetic wounds: a macrophage-centered view. Hyperglycemia/AGEs, oxidative stress and mitochondrial damage, and infection-associated signals sustain NF-κB activation and contribute to persistent inflammation and impaired wound repair. The lower panel summarizes representative macrophage-centered signaling pathways, regulatory defects, downstream inflammatory outputs, and amplification loops. Macrophages are depicted as key inflammatory amplifiers, but not as the only NF-κB-responsive cells in diabetic wounds. Solid and dashed arrows indicate direct signaling and indirect associations, respectively; red arrows indicate NF-κB-related outputs and inflammatory amplification. Created with BioRender.com (https://www.biorender.com/).

## 5 Engineered delivery of Chinese herbal medicine-derived therapeutics and NF-κB modulation

Chinese herbal medicine-derived therapeutics vary widely in chemical definition and compositional complexity, creating different demands for engineered delivery. For structurally defined compounds, formulation can be guided by known physicochemical properties, and drug loading and release can usually be quantified directly. As compositional and functional complexity increases, however, the therapeutic may contribute to material structure, contain multiple active constituents, or combine carrier and therapeutic functions. Chemical definition, compositional complexity, and functional role therefore provide a practical basis for comparing formulation requirements, release behavior, and the interpretation of biological effects across therapeutic systems.

### 5.1 Structurally defined small-molecule therapeutics

Many small molecules derived from Chinese herbal medicines have limited aqueous dispersibility, and some are susceptible to chemical degradation or washout by wound exudate. Nanostructuring, immobilization within material matrices, and molecular assembly have therefore been used to improve dispersibility or formulation stability and to control release.

#### 5.1.1 Alkaloids

Among alkaloids, engineered delivery has been studied mainly for berberine. Berberine is an isoquinoline alkaloid found in *Coptis chinensis*, *Phellodendron* species, and medicinal plants of the genus *Berberis* and has antibacterial, anti-inflammatory, antioxidant, and metabolic regulatory activities (Li et al., 2014; Neag et al., 2018). For wound application, its quaternary ammonium structure limits passive transmembrane transport, which poses a challenge for effective local delivery (Murakami et al., 2023).

Panda et al. encapsulated berberine in lecithin–chitosan nanoparticles with a mean diameter of 168.4 nm and an encapsulation efficiency of 82.3%. Compared with free berberine and blank nanoparticles, the formulation reduced inflammatory cell infiltration and promoted fibroblast proliferation, angiogenesis, and mature collagen deposition (Panda et al., 2021). Beyond nanoscale encapsulation, Zhang et al. dispersed berberine nanocolloids within a Ca^2+^-crosslinked hydrogel, which provided further control over release *in vitro*. Treatment increased SIRT1 expression and reduced total NF-κB, TNF-α, and IL-6 expression, accompanied by improved angiogenesis and tissue remodeling (Zhang et al., 2020). A different strategy used radially aligned fibers to combine directional guidance of fibroblast migration with gradual berberine release for nearly 1 month *in vitro*. The fibers also reduced macrophage production of IL-6 and TNF-α and promoted wound repair (Wang Q. et al., 2024). A more compositionally complex system immobilized berberine-modified ZnO nanocolloids within an injectable hydrogel, thereby combining berberine with another active component. The formulation increased Nrf2, HO-1, and NQO1 expression, reduced pro-inflammatory mediators, and promoted diabetic wound closure (Yin et al., 2022). Because a ZnO hydrogel-only control was not included, these effects should be attributed to the composite formulation as a whole.

Together, these studies illustrate distinct engineering functions of different berberine delivery platforms. The lecithin–chitosan nanoparticle study quantified particle size and encapsulation efficiency, whereas the Ca^2+^-crosslinked hydrogel and aligned-fiber systems primarily demonstrated changes in release profiles *in vitro* or wound-interface function. None directly quantified berberine exposure within wound tissue. NF-κB-related evidence also differed across formulations: the Ca^2+^-crosslinked hydrogel study measured total NF-κB expression rather than a core activation event, whereas the other systems mainly reported inflammatory or antioxidant responses.

#### 5.1.2 Flavonoid and non-flavonoid polyphenols

Flavonoid and non-flavonoid polyphenols are generally incorporated into engineered systems as drug cargoes. Their formulation requirements vary with solubility, chemical stability, and interactions with the carrier matrix. Curcumin is a major diarylheptanoid polyphenol in turmeric rhizomes and has anti-inflammatory and antioxidant activities. Its poor aqueous solubility and susceptibility to degradation under light and alkaline conditions make it difficult for the free compound to maintain stable local activity (Anand et al., 2007; Hewlings and Kalman, 2017). Li et al. encapsulated curcumin in chitosan nanoparticles, which showed slower drug release and increased macrophage uptake *in vitro*. In diabetic rats, the formulation reduced wound inflammation and promoted angiogenesis (Li et al., 2019). Liao et al. loaded curcumin into aligned poly (lactic-co-glycolic acid) (PLGA) fibers and grafted heparin onto the fiber surface. Fiber alignment supported cell migration, curcumin was released in a sustained manner, and surface-bound heparin helped enrich endogenous growth factors at the wound site. Treatment increased glutathione peroxidase expression and reduced NF-κB mRNA levels in wound tissue, accompanied by improved re-epithelialization, angiogenesis, and collagen deposition (Liao et al., 2021).

Quercetin is widely distributed in medicinal plants such as *Sophora japonica* and has antioxidant and anti-inflammatory activities (He et al., 2016; Alizadeh et al., 2023). Liu et al. encapsulated quercetin in chitosan nanoparticles with a mean diameter of 222.0 ± 7.57 nm and an encapsulation efficiency of 98.55% ± 0.56%, and then embedded the nanoparticles in a gelatin methacryloyl (GelMA) hydrogel. Free quercetin was completely released within 8 h, whereas release from the composite hydrogel reached a plateau after 48 h. The composite reduced RAGE and total NF-κB protein expression, as well as ROS and pro-inflammatory mediators, while favoring a reparative macrophage state and promoting angiogenesis (Liu et al., 2026). The study also evaluated *ex vivo* skin permeation, but did not directly quantify quercetin exposure within the wound. Taxifolin, also known as dihydroquercetin, occurs in medicinal plants including larch and milk thistle. Wang et al. incorporated taxifolin into sodium alginate/poly (vinyl alcohol) (PVA) electrospun fibers. The resulting fibrous membrane showed a continuous morphology and a cumulative *in vitro* release of 64.6% ± 3.7% over 24 h. Western blot analysis showed lower levels of proteins associated with TLR4/NF-κB/NLRP3 signaling and higher VEGFA and PDGFA expression, accompanied by reduced inflammation and accelerated tissue repair (Wang et al., 2023). Across these polyphenol systems, NF-κB-related evidence remained expression-based rather than activation-based: curcumin was assessed at the mRNA level, quercetin at the level of total NF-κB protein, and taxifolin through pathway-associated protein changes. None of these studies evaluated IκBα degradation or p65 nuclear translocation.

#### 5.1.3 Terpenoids and saponins

Some terpenoids and saponins serve not only as therapeutic cargoes but also as building blocks for material assembly. Glycyrrhizic acid is found in the dried roots and rhizomes of licorice. Its hydrophilic sugar chains and hydrophobic triterpenoid scaffold confer amphiphilic properties that enable self-assembly through noncovalent interactions (Selyutina and Polyakov, 2019; Li et al., 2020; Tucker et al., 2021). Qian et al. used Zn^2+^ to induce the assembly of glycyrrhizic acid into a supramolecular network, which was combined with photocrosslinked methacrylated silk fibroin to form an interpenetrating network hydrogel. Glycyrrhizic acid contributed to network formation, while the silk fibroin component improved structural stability. The hydrogel was injectable and conformed to the wound. Treatment also modulated macrophage states and promoted the transition from the inflammatory to the reparative phase (Qian et al., 2022). Li et al. incorporated glycyrrhizic acid–Zn^2+^ assemblies into a methacrylated konjac glucomannan network, further improving hydrogel stability. The resulting system reduced ROS accumulation and TNF-α expression in the wound, shifted macrophages toward a reparative state, and improved re-epithelialization, angiogenesis, and collagen deposition (Li J. et al., 2025). In these systems, glycyrrhizic acid therefore functions as both a therapeutic component and a structural building block. Zn^2+^ participates in glycyrrhizic acid self-assembly and network formation, whereas the antioxidant and immunomodulatory effects were evaluated at the level of the final composite hydrogel.

Engineered delivery of ginsenoside Rg5 combines nanoscale formulation with conformability to wound geometry. Rg5 is a rare ginsenoside generated during the steaming of ginseng (Liu et al., 2021; Chen et al., 2023). Shen et al. loaded Rg5 into F127–polyethyleneimine nanoparticles and immobilized the particles within an oxidized polysaccharide–collagen fiber hydrogel through dynamic covalent bonds. The nanoparticles improved Rg5 dispersion and prolonged its release *in vitro*. The shape-remodeling hydrogel enabled the formulation to conform to and fill tunneling wounds. The formulation modulated glucose metabolism and immune-cell states within the wound and shortened the time to healing (Shen et al., 2023). The engineered formulation did not assess NF-κB-related endpoints. In a separate non-engineered study, Rg5 reduced p65 and SLC7A11 expression in diabetic wounds and improved dendritic-cell efferocytosis through direct binding to SLC7A11 (Xia et al., 2023). Paeoniflorin is a representative monoterpene glycoside found in *Paeonia lactiflora* and *Paeonia veitchii*. Guo et al. encapsulated paeoniflorin in ROS-responsive micelles containing thioketal linkages and immobilized the micelles within an aminated gelatin–oxidized dextran hydrogel through dynamic Schiff-base bonds. ZnO nanoparticles were incorporated as an additional antibacterial component. Acidic conditions promoted dissociation of the Schiff-base bonds, whereas ROS cleaved the thioketal linkages within the micelles, together regulating paeoniflorin release *in vitro*. The resulting composite hydrogel controlled wound infection and promoted angiogenesis and tissue repair (Guo et al., 2022). NF-κB-related evidence for paeoniflorin came from a separate non-engineered study, in which treatment reduced CXCR2, nuclear NF-κB, and phosphorylated IκB levels and inhibited NLRP3 inflammasome activation (Sun et al., 2021). Unlike glycyrrhizic acid, which directly contributes to network formation, Rg5 and paeoniflorin function primarily as therapeutic cargoes in these systems. The available pharmacological evidence also differs in depth: Rg5 was associated mainly with reduced p65 expression, whereas paeoniflorin was linked to changes in nuclear NF-κB and phosphorylated IκB levels. Whether these effects are retained or enhanced after engineering remains to be tested directly in the corresponding formulations.

#### 5.1.4 Quinones and phenylpropanoids

Quinones and phenylpropanoids present different physicochemical constraints, leading to distinct priorities in delivery design. Rhein is an anthraquinone constituent of rhubarb and other medicinal herbs. Rhein can form self-assembled structures through noncovalent interactions (Zheng et al., 2019). Zhao et al. assembled rhein into fibrous structures and encapsulated them within a hydrogel formed through host–guest interactions between ferrocene and β-cyclodextrin. Oxidation of ferrocene by ROS weakened the host–guest interactions, leading to hydrogel dissociation and release of the self-assembled rhein structures. Compared with free rhein, the responsive system helped preserve the integrity of the rhein assemblies and reduced ROS accumulation and excessive inflammation in the wound (Zhao et al., 2020). A later design combined the rhein self-assembled network with photocrosslinked GelMA to form a double-network hydrogel with greater mechanical support. Calcitonin gene-related peptide (CGRP) was further incorporated into this system to generate CGRP@Rh-GelMA. GelMA improved the mechanical stability and wet-tissue adhesion of the hydrogel. *In vitro* studies showed release of both rhein and CGRP for approximately 10 days. Compared with GelMA alone, Rh-GelMA reduced wound ROS and NET-associated markers, including MPO and CitH3. Addition of CGRP was accompanied by further improvement in reparative macrophage states and angiogenesis. Proteomic analysis of wounds treated with the final composite hydrogel showed negative enrichment of NF-κB- and NET-related pathways (Zhou et al., 2026).

Cinnamaldehyde is a major phenylpropanoid constituent of cinnamon essential oil. Its high volatility makes it susceptible to loss during local application (Yildiz et al., 2019). Lin et al. incorporated cinnamaldehyde into an MMP-9-responsive injectable hydrogel that released the compound progressively under conditions of elevated MMP-9. Cinnamaldehyde activated transient receptor potential ankyrin 1 (TRPA1), promoting Ca^2+^ influx and phosphorylation of Ca^2+^/calmodulin-dependent protein kinase II (CaMKII), followed by increased Nrf2 nuclear translocation and glutathione peroxidase 4 (GPX4) expression (Lin et al., 2025). A separate non-engineered study showed that cinnamaldehyde attenuated AGEs/RAGE signaling and reduced phosphorylation of IκBα and p65 (Yu et al., 2025).

### 5.2 Chinese herbal polysaccharides

Chinese herbal polysaccharides can provide biological activity while also contributing to material construction. Their abundant hydroxyl groups support oxidation, grafting, and crosslinking, enabling them to form hydrogel networks or serve as components of nanocarriers and microneedle matrices. This dual role is formulation-dependent: some polysaccharides act as both structural and bioactive components, whereas others mainly support the delivery of additional therapeutics. Their material and biological contributions therefore need to be interpreted together.

*Ganoderma lucidum* polysaccharides, obtained from the fruiting bodies, mycelia, or spores of *Ganoderma*, consist mainly of β-glucans and heteropolysaccharides. Source and extraction conditions influence their composition, molecular weight, and degree of branching, with consequences for both biological activity and material-forming properties (Kou et al., 2023). Li et al. oxidized *G. lucidum* polysaccharides, yielding an aldehyde content of 1.33 ± 0.05 mmol/g and a degree of oxidation of 32.28% ± 2.55%. The aldehyde groups formed dynamic Schiff-base bonds with carboxymethyl chitosan, and sodium alginate was added to produce a double-network hydrogel. In this design, the oxidized polysaccharides served as both a network-forming polymer and a bioactive component, while the double-network architecture improved structural stability and tissue adhesion. Under high-glucose conditions, the hydrogel reduced ROS levels, promoted a shift of macrophages toward a reparative state, and improved fibroblast proliferation and migration. *In vivo*, treatment reduced wound inflammation and restored the epidermis, skin appendages, and collagen architecture (Li F. et al., 2024). *Bletilla striata* polysaccharide is a water-soluble glucomannan from the tubers of *B. striata*, composed mainly of mannose and glucose. Its hydrophilicity, film-forming capacity, and hemostatic and tissue-repair-promoting activities support its use in wound formulations, although it does not readily form a mechanically stable covering on its own (Chen et al., 2018).

Liu et al. linked *Astragalus membranaceus* polysaccharide and baicalein through phenylboronic acid to form ROS-responsive nanoparticles carrying berberine, and then incorporated the particles into *B. striata* polysaccharide/carboxymethyl chitosan microneedles (Liu et al., 2024). In this system, *B. striata* polysaccharide formed the dissolving microneedle matrix, *A. membranaceus* polysaccharide and baicalein formed the responsive carrier, and berberine was the encapsulated cargo. The microneedles penetrated the biofilm-covered wound surface and dissolved, while the high-ROS environment promoted nanoparticle disassembly and release of the active components. Treatment reduced wound ROS levels and p65 phosphorylation, decreased TNF-α and IL-6 expression, and increased angiogenesis and collagen deposition. Because multiple bioactive components contributed to the formulation, these NF-κB-related changes reflect the effect of the composite microneedle system as a whole. These formulations show how the role of a herbal polysaccharide changes with system design. Oxidized *G. lucidum* polysaccharide was both network-forming and biologically active, whereas *B. striata* and *A. membranaceus* polysaccharides primarily formed the dissolving matrix and ROS-responsive carrier, respectively.

### 5.3 Single-herb extracts and active fractions

Single-herb extracts and active fractions contain multiple chemical constituents that differ in solubility and stability. These differences complicate uniform dispersion and simultaneous local delivery. Nanoemulsions and hydrogels can improve dispersion or modulate release, while three-dimensional (3D) printing adds control over scaffold geometry and wound conformity.

*Poria cocos* triterpenes are a triterpenoid-rich active fraction obtained from the sclerotia of *P. cocos* and consist mainly of lanostane-type triterpenes and their derivatives (Nie et al., 2020). Representative constituents such as pachymic acid have low aqueous solubility, which limits their uniform dispersion in aqueous formulations (Wei et al., 2022). Ding et al. prepared an oil-in-water nanoemulsion of the triterpene fraction and incorporated it into a hyaluronic acid hydrogel. The nanoemulsion improved dispersion stability, while the hydrogel provided a uniform, porous, and elastic matrix. In diabetic rats, the formulation reduced wound inflammation, shifted macrophages toward an M2-like reparative state, and promoted re-epithelialization, collagen deposition, and angiogenesis (Ding et al., 2023). NF-κB-related evidence came from a separate study of the unformulated triterpene fraction. In THP-1 cells, the fraction reduced NF-κB, IL-1β, IL-6, and TNF-α mRNA expression and favored an M2-like macrophage state. In endothelial cells, it increased PI3K and Akt phosphorylation and improved migration and angiogenesis-related functions (Ding et al., 2024). *Scutellaria baicalensis* extract is rich in flavonoids such as baicalin and baicalein (Wang et al., 2018). Zhu et al. incorporated the extract into a hydrogel formed through dynamic imine bonds between oxidized sesbania gum and carboxymethyl chitosan. The porous, self-healing hydrogel retained free-radical-scavenging activity, and cumulative extract release reached a plateau of 68.8% ± 2.1% at 12 h in PBS. In *Staphylococcus aureus*-infected diabetic wounds, the extract-loaded hydrogel improved wound closure, granulation tissue formation, epidermal regeneration, and collagen deposition relative to both free extract and blank hydrogel (Zhu et al., 2024).

Three-dimensional printing can match drug-loaded scaffold geometry to irregular wounds (Zhou et al., 2024). *Centella asiatica* extract is rich in triterpenoids such as asiaticoside, madecassoside, asiatic acid, and madecassic acid and has anti-inflammatory and tissue-repair-promoting activities (Witkowska et al., 2024). Wang et al. incorporated *Centella asiatica* extract into a gelatin/sodium alginate bioink to fabricate a 3D-printed scaffold. The scaffold could be shaped to fit the wound and served as a local carrier for the extract. In chronic diabetic wounds, the scaffold modulated macrophage and local immune responses and promoted wound closure and tissue reconstruction (Wang X. et al., 2024). NF-κB-related evidence came from a separate non-engineered study. In AGE-stimulated macrophages, *Centella asiatica* treatment was associated with lower p-NF-κB levels and reduced NO, IL-1β, IL-6, and TNF-α production. Local treatment also reduced inflammatory infiltration and increased collagen deposition and angiogenesis in diabetic wounds (Xiao et al., 2025).

These studies demonstrate distinct engineering gains: improved dispersion for the *P. cocos* fraction, measured release and a formulation-level benefit for *Scutellaria baicalensis* extract, and geometric adaptability for the *Centella asiatica* scaffold. Only the *Scutellaria baicalensis* study compared the engineered formulation with both free extract and blank material, but it did not assess core NF-κB activation. For *P. cocos* and *Centella asiatica*, NF-κB-related effects were evaluated only in studies without engineered carriers. Whether delivery enhances these effects therefore remains unknown.

### 5.4 Local pharmacological effects and engineered delivery of Chinese herbal formulas

Among conventional topical preparations, Shengji Huayu Formula gel reduced NF-κB p50 nuclear staining in the epidermis at the wound edge on day 15 and promoted re-epithelialization (Kuai et al., 2018). Topical Pien Tze Huang reduced the nuclear-to-cytoplasmic p65 ratio and increased IκBα and RelB expression, accompanied by lower NKG2D, NOD2, TLR2, IFN-γ, and IL-1β levels (Cao et al., 2023). Huhuang Decoction reduced p65 phosphorylation and inflammatory cytokine levels while increasing HIF-1α, VEGF, and CD31 expression (Zhang et al., 2025). Conventional medicated oils and ointments have also shown NF-κB-related regulatory effects. Compound Tongye Burn Oil uses sesame oil as both the extraction medium and topical matrix. Treatment altered markers associated with IL-17/NF-κB/AP-1 signaling, reduced wound inflammation, and restored tight-junction protein expression, thereby promoting skin-barrier reconstruction and wound closure (Lu et al., 2025). In IgE/DNP-stimulated mast cells, Zizhu Ointment reduced the phosphorylation of LYN, SYK, PLCγ, IKKα/β, and p65 and suppressed mast-cell degranulation (Sun et al., 2026). Together, these studies support the capacity of topical Chinese herbal formulas to modulate NF-κB-related signaling.

More recent studies have incorporated multi-herb extracts into hydrogels, nanoyarn dressings, and microneedles. Chen et al. dispersed micronized Huoxue Tongluo Formula extract in a photocrosslinked hydrogel composed of methacrylated sesbania gum and γ-polyglutamic acid. The interpenetrating network improved mechanical properties and extract dispersion. In infected diabetic wounds, the complete formulation reduced bacterial burden, ROS, and inflammatory infiltration and promoted epidermal regeneration, collagen deposition, and angiogenesis (Chen et al., 2025). Li et al. electrospun extracts of the *Salvia miltiorrhiza–Pueraria lobata* herb pair with gelatin/polylactic acid to form nanoyarns and wove them into warp–weft dressings. The resulting dressing combined mechanical strength and surface wettability and provided a fibrous interface that supported fibroblast adhesion and proliferation. The extract-loaded dressings also reduced inflammatory mediator release from pro-inflammatory-like macrophages and promoted re-epithelialization, angiogenesis, and hair-follicle regeneration (Li Y. et al., 2024). Tang et al. incorporated dragon’s blood and *S. miltiorrhiza* extracts into dissolving polyvinylpyrrolidone microneedles. Release of the representative constituents dracorhodin and salvianolic acid B was quantified over 6 h. Among the tested microneedle groups, the dual-extract formulation produced the fastest wound repair and improved wound microcirculation (Tang et al., 2024).

### 5.5 Engineered delivery of Chinese herbal medicine-derived nanovesicles and related biological nanoparticles

Chinese herbal medicine-derived nanovesicles can carry diverse bioactive substances, including lipids, proteins, nucleic acids, and small molecules, and facilitate their entry into target cells, providing a new modality for the integrated delivery of complex Chinese herbal medicine-derived therapeutics (Hao et al., 2024). Current studies encompass naturally isolated nanovesicles or exosome-like nanoparticles, mechanically generated vesicle mimetics, and other biological nanoparticles derived from Chinese herbal medicines. Because these particles differ in their mode of formation, membrane structure, and cargo composition, they also differ in stability, cellular uptake, and how they interface with carrier materials (Jo et al., 2025).

Naturally isolated Chinese herbal medicine-derived nanovesicles can act on wound cells as intact bioactive units. Tan et al. isolated exosome-like nanovesicles from ginseng roots and found that they could be taken up by vascular endothelial cells injured by high glucose. Following treatment, the expression of glycolysis-related genes, including *PFKM*, increased, accompanied by higher fructose-1,6-bisphosphate and lower acetyl-CoA levels, suggesting a shift in cellular energy metabolism toward glycolysis. Intracellular ROS levels decreased, while cell proliferation, migration, and tube-forming capacity recovered. *In vivo* experiments also showed increased angiogenesis within the wound (Tan et al., 2024). Exosome-like nanoparticles derived from *S. miltiorrhiza* similarly promoted fibroblast and endothelial-cell proliferation and reduced ROS, IL-1β, IL-6, and TNF-α levels in diabetic wounds (Bai et al., 2026). These studies support the intrinsic bioactivity of naturally isolated Chinese herbal medicine-derived particles, but do not determine whether incorporation into a carrier provides an additional delivery benefit.

Engineered carriers add distinct delivery functions to Chinese herbal medicine-derived nanoparticles and vesicle mimetics. Wu et al. incorporated turmeric-derived nanoparticles into a cellulose nanofiber/sodium alginate aerogel. The aerogel provided fluid absorption and conformability to wound geometry while improving the storage stability of the nanoparticles. The released turmeric-derived nanoparticles increased Nrf2, HO-1, and NQO1 expression in fibroblasts and reduced TLR4, MyD88, and pro-inflammatory mediator levels in macrophages, accompanied by reduced wound inflammation and improved tissue reconstruction (Wu et al., 2024). Wang et al. prepared *Aloe vera* peel-derived vesicle mimetics by high-pressure homogenization and then incorporated them into a hydrophobically modified hydroxypropyl methylcellulose hydrogel. Compared with Carbomer gel, this hydrogel better preserved particle structure, and *in vitro* release studies showed sustained release of the vesicle mimetics for 72 h. The formulation reduced total NF-κB protein, COX-2, and iNOS expression in LPS-stimulated macrophages and promoted re-epithelialization and collagen remodeling in diabetic mouse wounds, with better reparative effects than free vesicle mimetics or the blank hydrogel (Wang J. et al., 2026).

Responsive materials can couple the release of natural vesicles to the high-glucose or high-ROS environment of diabetic wounds. Yang et al. incorporated *Houttuynia cordata*-derived extracellular vesicles into a phenylboronic acid-modified recombinant type III collagen hydrogel. High glucose and ROS promoted hydrogel degradation and vesicle release. Treatment reduced IκBα phosphorylation, restored total IκBα levels, and concurrently decreased p65 phosphorylation, accompanied by lower levels of pro-inflammatory mediators and increased angiogenesis and collagen deposition in the wound (Yang et al., 2025). Wu et al. constructed an ROS-responsive bilayer hydrogel loaded with *Ginkgo biloba*-derived exosome-like nanovesicles. The outer network containing ε-polylysine and copper ions was designed to provide early antibacterial activity, whereas elevated ROS cleaved thioketal bonds in the inner hydrogel and triggered release of the *G. biloba*-derived vesicles. After infection was controlled, the final composite hydrogel reduced cellular injury and promoted cell proliferation and angiogenesis (Wu et al., 2026). The system enabled different active components to function at different stages, but the overall reparative effect should still be attributed to the bilayer hydrogel as a whole.

Chinese herbal medicine-derived nanovesicles can also be co-delivered with complementary bioactive particles. Zhang et al. co-loaded *H. cordata*-derived carbon dots and extracellular vesicles into a GelMA hydrogel. The carbon dots mainly inhibited bacteria and biofilms, whereas the vesicles were associated with reduced ROS levels, modulation of macrophage state, and improved fibroblast migration. The co-loaded hydrogel showed better antibacterial and reparative effects than hydrogels loaded with either the carbon dots or vesicles alone, supporting an additional benefit from combining the two *H. cordata*-derived active components (Zhang et al., 2026). Weng et al. co-loaded bitter melon-derived exosome-like nanovesicles and mesenchymal stem cell-derived exosomes into a GelMA/dopamine hydrogel. Both types of vesicles were released from the material in a sustained manner. Compared with the individually loaded systems, the co-loaded hydrogel showed stronger anti-inflammatory and antioxidant effects and further improved macrophage state and wound repair. Treatment was also associated with reduced AGE accumulation and increased angiogenesis and collagen deposition (Weng et al., 2025).

NF-κB-related evidence differs in depth across these Chinese herbal medicine-derived particle systems. Ginseng- and *S. miltiorrhiza*-derived particles were evaluated mainly through metabolic, oxidative, and inflammatory readouts. Turmeric-derived nanoparticles were associated primarily with Nrf2-and TLR4/MyD88-related changes, whereas *A. vera* peel-derived vesicle mimetics reduced total NF-κB protein without directly assessing NF-κB activation. By contrast, the *H. cordata* extracellular vesicle hydrogel reduced phosphorylation of both IκBα and p65, providing direct readouts of canonical NF-κB activation. These Chinese herbal medicine-derived particle types are also not structurally equivalent. Naturally isolated extracellular vesicles generally retain endogenous membrane structures and cargoes, whereas the composition of mechanically generated vesicle mimetics and other Chinese herbal medicine-derived biological nanoparticles is more strongly influenced by the preparation process. The relationships among engineered delivery, local exposure, NF-κB-related evidence, and wound repair are summarized in Figure 2.

**FIGURE 2:**
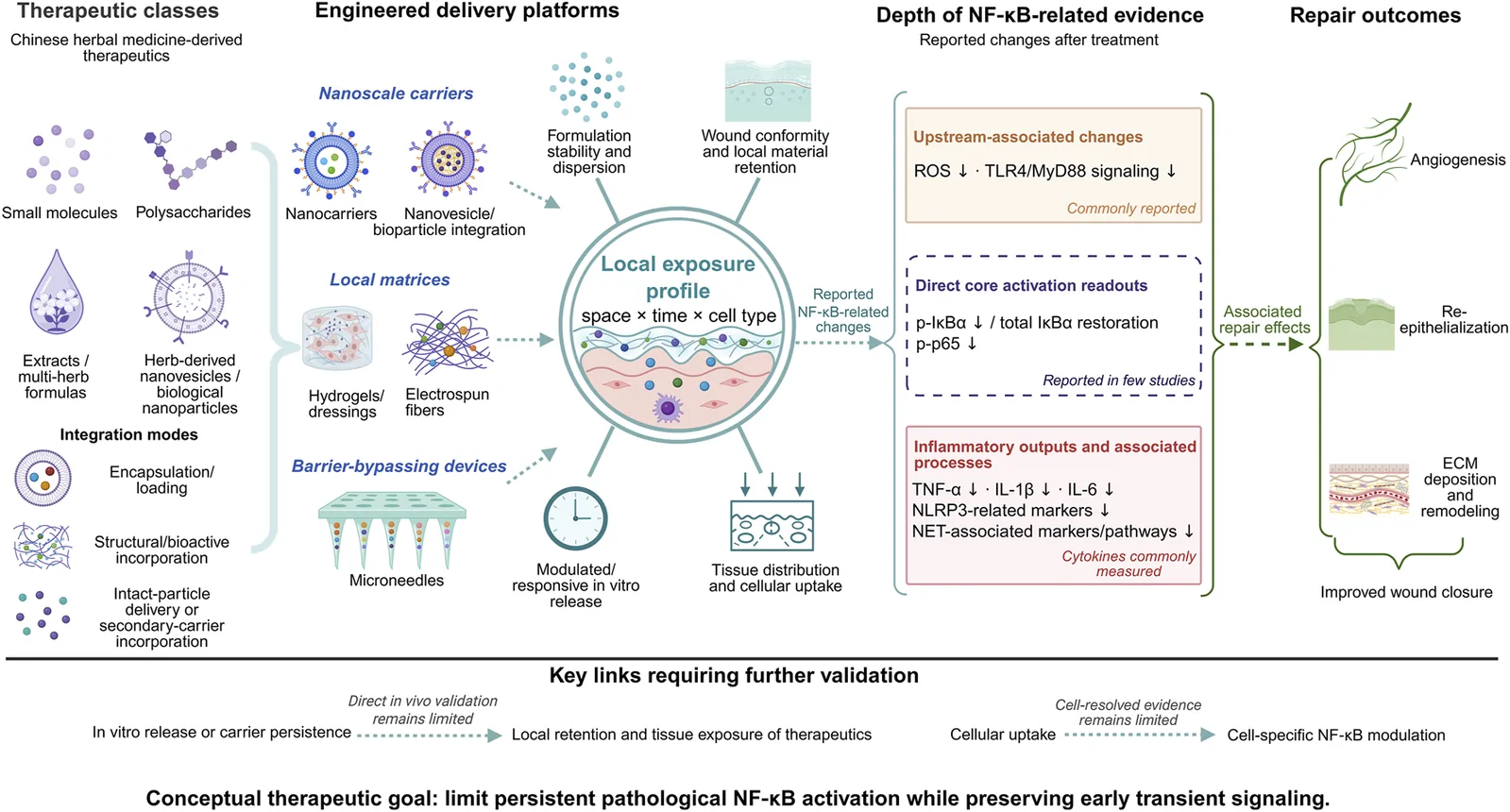
Engineered delivery, NF-κB-related evidence, and repair outcomes in diabetic wounds. Chinese herbal medicine-derived therapeutics are incorporated into nanoscale carriers, local matrices, or barrier-bypassing devices to shape local exposure and biological responses. Current studies more frequently report upstream or downstream NF-κB-related changes than direct core-activation readouts. Dashed teal arrows indicate incompletely validated links between delivery processes, local exposure, and NF-κB-related changes. The dashed green arrow denotes associated repair effects and does not establish causal mediation by NF-κB. Created with BioRender.com (https://www.biorender.com/).

## 6 Discussion: common patterns and translational pathways of NF-κB modulation through engineered delivery

### 6.1 Engineered delivery reshapes the functional state of therapeutics

Chinese herbal medicine-derived therapeutics can assume distinct functional states after incorporation into engineered systems. For many structurally defined small molecules, the therapeutic remains primarily a cargo, while engineering changes its formulation, release, or presentation at the wound interface. The berberine systems reviewed above illustrate this pattern: nanoparticle encapsulation, hydrogel incorporation, and radially aligned fibers modified delivery behavior while berberine remained the identifiable therapeutic cargo (Panda et al., 2021; Zhang et al., 2020; Wang Q. et al., 2024).

In other systems, the boundary between therapeutic and material is less distinct. Glycyrrhizic acid contributes directly to supramolecular assembly, rhein forms self-assembled structures, and oxidized *G. lucidum* polysaccharide participates in hydrogel network formation while retaining biological activity (Qian et al., 2022; Zhao et al., 2020; Li F. et al., 2024). In these cases, the assembled and released forms of the therapeutic may differ in diffusion behavior and cellular accessibility, so material construction becomes part of how the pharmacological entity is presented to the wound.

Extracts and multi-herb formulas introduce a different problem because their constituents can interact differently with the material matrix and may not be released in the same proportions. Current studies generally characterize either bulk extract release or a limited number of representative constituents, as in the *Scutellaria baicalensis* hydrogel and the dragon’s blood–*S. miltiorrhiza* microneedles (Zhu et al., 2024; Tang et al., 2024). Such measurements provide formulation- or release-related information but do not establish whether the relative composition reaching the wound is preserved.

Chinese herbal medicine-derived biological nanoparticles add another layer of complexity. Naturally isolated extracellular vesicles can act as intact bioactive units, whereas mechanically generated vesicle mimetics are shaped more directly by processing conditions; their incorporation into hydrogels or other carriers can further modify particle stability and release (Jo et al., 2025; Yang et al., 2025; Wang J. et al., 2026). Engineered delivery can therefore modify not only the release of a therapeutic but, in some systems, also the physical or compositional form in which the active entity is presented to the wound.

### 6.2 Evidence gaps across material performance, local exposure, and NF-κB modulation

The value of an engineered material depends on whether it addresses the delivery barrier it was designed to overcome. Because the systems reviewed above target different delivery problems, their engineering performance should be interpreted against the intended design objective rather than a uniform set of parameters.

Most studies demonstrate successful material fabrication and altered *in vitro* release behavior, but direct evidence for the actual fate of the therapeutic after delivery into the wound remains limited. Slower *in vitro* release does not necessarily mean that the therapeutic remains in the wound for longer, and persistence of the carrier or a fluorescent signal cannot substitute for direct measurement of the therapeutic itself. Compared with material characterization and release testing, far fewer studies directly assess local therapeutic levels, tissue distribution, or cellular uptake. An evidence gap therefore remains between material immobilization or delayed release and increased local exposure.

A similar evidence gap applies to NF-κB modulation. Many studies report reduced ROS levels, attenuated TLR4/MyD88 signaling, or lower levels of inflammatory mediators. These changes suggest an improvement in the NF-κB-related inflammatory environment but do not directly demonstrate inhibition of the core cascade. Changes in NF-κB mRNA or total protein levels are likewise insufficient to determine the activation state of the pathway. Fewer studies directly assess IκBα phosphorylation or degradation, p65 phosphorylation and nuclear translocation, and even fewer use specific inhibition, knockdown, or rescue experiments to verify an NF-κB-mediated effect. Current studies therefore more often document these evidence domains in parallel than connect them through direct measurements of local exposure and core NF-κB activation.

### 6.3 Temporal and cellular dimensions of NF-κB modulation

NF-κB activity is inherently dynamic, and p65 phosphorylation or nuclear translocation measured at a single endpoint cannot adequately capture its full temporal behavior. In a cellular model, Tabata et al. found that IκBα knockdown shifted TNF-α-induced nuclear NF-κB activity from an oscillatory response to a sustained state and increased inflammatory gene expression (Tabata et al., 2024). The biological meaning of an NF-κB-related change therefore depends on its temporal context, not simply on whether a marker increases or decreases. In studies of engineered formulations, a lower NF-κB-related readout at a single time point cannot distinguish attenuation of persistent pathological activation from broader suppression of NF-κB signaling, including responses that may be required during early host defense. Because most studies assess only one or a few time points, this distinction remains unresolved.

The effects of NF-κB also depend on the cell type and functional state in which the pathway is active. Single-cell transcriptomic analysis of diabetic foot ulcers has revealed marked functional differences among endothelial-cell subsets. Subsets associated with non-healing showed greater enrichment of inflammatory and TNF signaling, whereas healing-associated subsets exhibited distinct metabolic features (Zhao et al., 2025). Cells within the wound therefore do not respond uniformly to inflammatory stimuli. Accordingly, changes in p65 or inflammatory mediators measured in whole wound tissue may reflect altered signaling within individual cells, shifts in cellular composition, or both. Whole-tissue measurements alone cannot reliably distinguish among these possibilities. Engineered delivery adds another layer of variability because local retention, tissue distribution, and cellular uptake determine where the Chinese herbal medicine-derived therapeutic is present and for how long. These exposure patterns are shaped by material release, wound exudate, material degradation, tissue binding, and local clearance. It therefore remains unclear which exposure profile is most appropriate for modulating persistent pathological NF-κB activation without broadly suppressing its phase- and cell-dependent functions. Future studies should assess local exposure and NF-κB activity together across relevant time points and cell populations rather than treating prolonged release as a therapeutic endpoint in itself.

### 6.4 Therapeutic attribution and system complexity

Attribution becomes particularly difficult when Chinese herbal medicine-derived therapeutics are combined with bioactive carriers or additional active components. Comparing the free therapeutic with the loaded formulation helps determine whether engineering provides an additional benefit, while a blank-carrier control helps identify the contribution of the material itself. Systems containing additional active components, such as ZnO, bioactive peptides, copper ions, or vesicles from non-herbal sources, require component-specific controls to distinguish their individual contributions. Without such controls, NF-κB-related changes and wound-healing outcomes should be attributed to the final formulation rather than to a single Chinese herbal medicine-derived therapeutic.

As delivery systems combine antibacterial and antioxidant activities, stimuli-responsive release, and pro-angiogenic functions, their complexity increases. Additional functions do not necessarily provide additional therapeutic benefit and may increase component interactions, preparation variability, and difficulties in quality control. Greater complexity should therefore be justified by its ability to address a defined delivery or therapeutic barrier and to provide a demonstrable advantage over a simpler system.

### 6.5 Quality control, biocompatibility, and model relevance

Variability in the source and composition of Chinese herbal medicine-derived therapeutics can propagate through extraction, assembly, and formulation. This issue is particularly relevant to polysaccharides, extracts, and biological nanoparticles, because differences in composition, molecular weight, or macromolecular structure can affect encapsulation, crosslinking, interfacial interactions, and release. Quality control should therefore characterize both the Chinese herbal medicine-derived therapeutic and the final engineered formulation, including relevant compositional attributes, material structure, release behavior, and biological activity.

Safety evaluation should focus on the final formulation rather than on individual components alone. ZnO, copper ions, photoinitiators, crosslinking reactions, and responsive degradation can introduce interactions or products that are not captured by separate component testing. Repeated or prolonged wound exposure further makes degradation products, local clearance, and tissue responses relevant to practical use. Sterilization, storage, and scale-up may also alter material networks, particle states, and release behavior, so properties measured under laboratory conditions cannot be assumed to persist in the final product.

Chinese herbal medicine-derived biological nanoparticles require additional quality controls because their identity depends strongly on source and preparation. Source material, isolation, and storage can alter the composition and stability of naturally isolated extracellular vesicles, whereas mechanically generated vesicle mimetics are more directly influenced by processing conditions (Kim et al., 2022). Particle size and morphology alone are therefore insufficient to define these systems. Quality assessment should also establish their composition, formation process, relevant structural characteristics, and stability during storage.

Model relevance should be judged against the therapeutic barrier that an engineered formulation is intended to address. Conventional diabetic skin-defect models capture delayed repair under diabetic conditions, whereas infected, ischemic, or biofilm-containing models are more appropriate for systems designed to target these additional pathological factors. No single animal model reproduces the combined neuropathy, ischemia, infection, and repeated mechanical loading of the human diabetic foot. Faster wound closure therefore demonstrates efficacy within a particular model rather than direct clinical translatability. The key question is whether the chosen model tests the barrier targeted by the engineered system and whether its advantage persists under more clinically relevant conditions.

## 7 Conclusion

Engineered delivery expands the local application of Chinese herbal medicine-derived therapeutics in diabetic wounds by adapting formulation and delivery to the physicochemical and functional characteristics of different therapeutic entities. Across studies, NF-κB-related evidence ranges from changes in upstream stimuli and downstream inflammatory outputs to a smaller number of studies assessing core activation events such as IκBα or p65 phosphorylation. The therapeutic goal should be attenuation of persistent pathological NF-κB activation rather than continuous pathway suppression, but current studies rarely resolve this distinction across wound-healing phases and cell types. Overall, current evidence more consistently supports improvements in formulation performance and wound repair than a delivery-dependent enhancement of NF-κB modulation. Future studies should connect local exposure, temporally and cellularly resolved NF-κB readouts, and wound outcomes within the same experimental framework, while using appropriate controls to distinguish therapeutic, carrier, and additional component effects.
